# Exploring the Correlation between Systemic Inflammatory Markers and Carotid Atherosclerosis Indices in Middle-Aged Adults: A Cross-Sectional Study

**DOI:** 10.3390/jcdd11030073

**Published:** 2024-02-21

**Authors:** Ji-Eun Song, Ji-In Hwang, Hae-Jin Ko, Ji-Yeon Park, Hee-Eun Hong, A-Sol Kim

**Affiliations:** 1 Department of Family Medicine, Kyungpook National University Chilgok Hospital, Daegu 41404, Republic of Korea; love2uje@naver.com (J.-E.S.); hwangjiin23@naver.com (J.-I.H.); 2 Department of Family Medicine, Kyungpook National University Hospital, Daegu 41944, Republic of Korea; liveforme@knu.ac.kr (H.-J.K.); miniev@naver.com (J.-Y.P.); hhe8824@naver.com (H.-E.H.); 3 Department of Family Medicine, School of Medicine, Kyungpook National University, Daegu 41944, Republic of Korea

**Keywords:** carotid stenosis, carotid plaque, inflammatory marker, neutrophil–lymphocyte ratio, platelet–lymphocyte ratio

## Abstract

Background: This study investigated the association between atherosclerosis and systemic inflammation markers, specifically the C-reactive protein (CRP), erythrocyte sedimentation rate (ESR), neutrophil-to-lymphocyte ratio (NLR), and platelet-to-lymphocyte ratio (PLR), in healthy middle-aged adults. Methods: A retrospective cross-sectional study was conducted on a total of 1264 Korean adults aged 40–65. We assessed these inflammatory markers and carotid metrics, such as carotid intima–media thickness (cIMT), plaque number (PN), plaque stenosis score (PSS), and plaque score (PS), using linear regression, logistic regression, and receiver operating characteristic analysis. Results: In males, the ESR and CRP were significantly correlated with the PN (*p* < 0.001 and *p* = 0.048, respectively). The ESR was correlated with the PN in females (*p* = 0.004). The NLR and PLR both correlated with the PS in males (*p* < 0.001 and *p* = 0.015, respectively) and females (*p* = 0.015 and *p* = 0.023, respectively). The odds ratio for the NLR as a risk factor for increased cIMT was 1.15 (95% confidence interval [CI], 1.03–2.15) for males and 1.05 (95% CI, 1.01–1.29) for females. The AUC for the NLR and PLR as a predictor for the PS showed significance in both men and women. Conclusions: Inflammatory markers, particularly the NLR and PLR, demonstrate a correlation with carotid atherosclerosis. Both the NLR and PLR hold potential as valuable surrogate markers for carotid atherosclerosis. To further substantiate their predictive efficacy, further prospective studies are needed.

## 1. Introduction

Cardiovascular diseases (CVDs) are globally recognized as the leading causes of disability and premature mortality [1]. Atherosclerosis is the main pathological process in CVDs, particularly ischemic heart disease and stroke [2,3]. Inflammatory and immune mechanisms play a pivotal role in the development and progression of atherosclerotic arteries [4]. Atherosclerosis is initiated by endothelium dysfunction derived from major contributors like elevated serum LDL-cholesterol, oxidative stress, mechanical stress, and inflammatory response, which play an important role in all stages of atherosclerosis [5,6]. Atherosclerotic plaques cause the blood vessel lumen to narrow, thicken, and harden, making them significant risk factors for ischemic stroke and transient ischemic attack [1,7].

Various inflammatory markers, including the erythrocyte sedimentation rate (ESR), C-reactive protein (CRP), and fibrinogen, have been explored to monitor the potential presence of atherosclerotic lesions and cardiovascular risk. Recent research has indicated that white blood cell (WBC) counts and their subtypes are reliable indicators of inflammation [8].

Previous studies have highlighted the neutrophil-to-lymphocyte ratio (NLR) and platelet-to-lymphocyte ratio (PLR) as readily available and cost-effective prognostic indicators [9,10]. The NLR provides insights into inflammatory conditions, including neutrophil elevation, lymphopenia, and lymphocyte apoptosis. Its role as an independent prognostic factor in coronary artery disease is well documented [11]. Furthermore, the PLR offers insight into aggregation and inflammatory pathways, and an elevated PLR value indicates the severity of coronary atherosclerotic disease [12].

There is evidence of a relationship between systemic inflammatory markers and atherosclerosis in older adults [13]. The association of the NLR and PLR with atherosclerosis is likely due to the activation of neutrophils and platelets in the plaque, leading to the progression of vascular wall lesions by inflammation and protein hydrolysis [13]. However, the association of these inflammatory markers with relatively healthy young patients remains uncertain. Atherosclerosis has evolved over many years, and aging is a recognized independent risk factor for both sexes [14,15]. Screening for asymptomatic with subclinical atherosclerosis in middle age is crucial to prevent unforeseen disabilities or death. Our study aims to explore the relationship between inflammatory markers and the carotid atherosclerosis index and evaluate their potential as predictive tools in relatively healthy adults.

## 2. Materials and Methods

### 2.1. Study Subjects

This retrospective cross-sectional study utilized data from 33,110 subjects who underwent health check-ups between 30 November 2018 and 31 December 2020 at Kyungpook National University Hospital and Kyungpook National University Chilgok Hospital in Korea. The cohort consisted of adults aged 40–65 who completed questionnaires about their sociodemographic background and past medical history and underwent carotid ultrasonography and laboratory tests.

**Figure 1:**
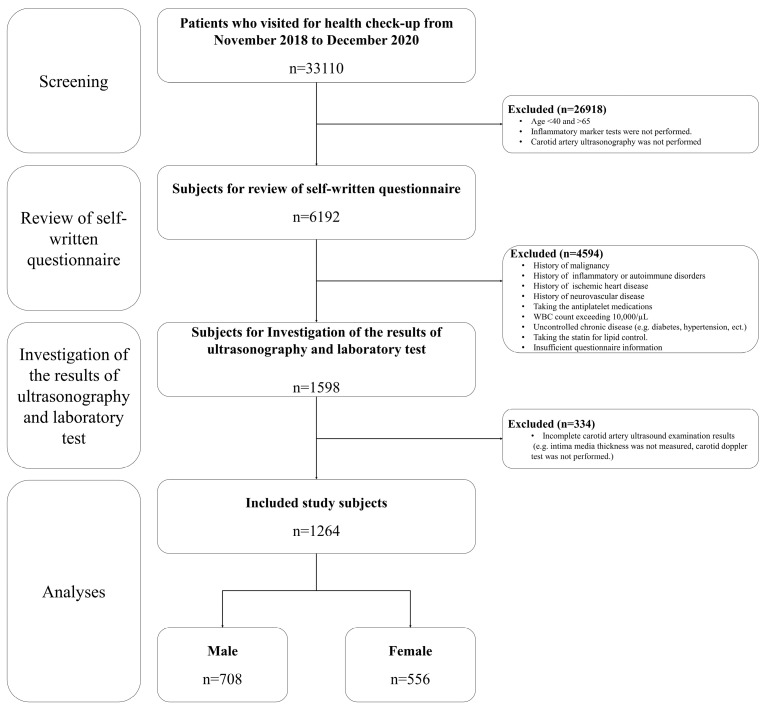
Study enrolment. Inclusion and exclusion process. WBC, white blood cell.

Participants were excluded if they had a history of malignancies or were diagnosed with inflammatory or autoimmune disorders, such as rheumatoid arthritis, osteoarthritis, ankylosing spondylitis, or Crohn’s disease. Additionally, those with ischemic heart disease, cerebrovascular afflictions, or uncontrolled chronic conditions with hypertension, diabetes, or dyslipidemia were not considered for inclusion. Participants on statin use to control their lipid profile were excluded. The study also excluded individuals on specific medications, such as antiplatelet agents (e.g., aspirin) or anti-thrombotic agents, as well as those with a WBC count exceeding 10,000/µL. Participants with incomplete data from questionnaires or lacking comprehensive carotid artery ultrasound results were further excluded. After applying these exclusion criteria, the final cohort consisted of 1264 subjects, encompassing 708 males and 556 females (Figure 1).

The study protocol received approval from the Institutional Review Board of Kyungpook National University Chilgok Hospital (IRB No. 2021-09-010). Due to the retrospective nature of the study, the requirement for patient consent was waived.

### 2.2. Demographics and Anthropometrics

Demographic data, including age, sex, comorbidities (such as hypertension, diabetes, and dyslipidemia), alcohol consumption, smoking habits, and exercise patterns, were collected using a health screening questionnaire.

Hypertension was noted if the patient had a physician-diagnosed condition, was on antihypertensive medication, or recorded a blood pressure of ≥140/90 mmHg. Diabetes mellitus was recognized if diagnosed by a physician, the patient was on anti-diabetic medication, or a fasting plasma glucose level of ≥126 mg/dL was detected. Dyslipidemia was marked if a patient was on cholesterol-lowering medication or if certain cholesterol levels were identified, such as total cholesterol ≥ 200 mg/dL, high-density lipoprotein (HDL) cholesterol < 45 mg/dL in males or <50 mg/dL in females, low-density lipoprotein (LDL) cholesterol ≥ 130 mg/dL, or triglycerides ≥ 150 mg/dL [16].

For alcohol consumption, individuals were categorized as non-drinkers or drinkers based on the National Institute on Alcohol Abuse and Alcoholism criteria. A drinker was defined as an individual consuming over 14 standard drinks per week for males and 7 for females. Regarding smoking, non-smokers were those who never smoked, former smokers had smoked less than 10 packs-years (py), and current smokers had smoked more than 10 py. Regular physical activity was defined as moderate–intensity exercise exceeding 150 min weekly.

Anthropometric measurements were conducted by skilled personnel using standardized methods. Height was recorded to the nearest 0.1 cm and weight to the nearest 0.1 kg, with participants in light clothing and without shoes. The Body Mass Index (BMI) was determined by dividing weight by the square of height (kg/m^2^). Blood pressure was taken after a 10 min seated rest using validated electronic devices.

### 2.3. Systemic Inflammatory Markers and Laboratory Tests

Blood samples were drawn from peripheral veins after participants fasted for a minimum of 8 h. The ADVIA 2120i hematology system (SIEMENS Healthineers, Forchheim, Germany) was utilized to measure WBC, neutrophil, lymphocyte, and platelet counts. The NLR was computed as the absolute neutrophil count divided by the absolute lymphocyte count, while the PLR was determined by dividing the platelet count by the absolute lymphocyte count. Assays for total cholesterol, triglyceride, low-density lipoprotein (LDL) cholesterol, high-density lipoprotein (HDL) cholesterol, fasting blood sugar, hemoglobin A1c (HbA1c), high-sensitivity CRP (hsCRP), and ESR were conducted using the CellDyn4000 (Abbott Laboratories, Chicago, IL, USA), Cobas 8000 c702 (Roche Diagnostics, Rotkreuz, Switzerland), and TEST1 (Alifax, Polverara, Italy).

### 2.4. Carotid Ultrasonography

Extracranial carotid duplex ultrasonography was conducted by three radiologists using three machines: the PHILLIPS IU-22, PHILLIPS EPIQ 7G, and the TOSHIBA APOLIO 500 (TUS-A500). All procedures were overseen by a senior specialist with extensive training in carotid artery ultrasonography from the Department of Radiology. For each participant, measurements were taken for the mean and max carotid intima–media thickness (cIMT) of the common carotid artery (CCA), peak systolic velocity (PSV) of the internal carotid artery (ICA), plaque presence in the carotid artery, and stenosis of the ICA. Additionally, the plaque number score (PN), plaque stenosis score (PSS), and carotid artery plaque score (PS) were utilized to assess carotid atherosclerosis.

Carotid ultrasound parameters were measured following the guidelines of the Mannheim carotid IMT and plaque consensus as well as the American Society of Echocardiography protocols. Before undergoing carotid ultrasonography, participants were advised to rest in a supine position for approximately 20 min. A pillow was positioned under the patient’s neck to facilitate hyperflexion. Subsequent scans were then conducted sequentially, covering the bilateral common carotid artery, carotid bifurcation, and external and internal carotid arteries. The cIMT was gauged in B-mode from longitudinal views of both the left and right common carotid arteries. The cIMT was averaged across measurements taken from the CCA wall without atherosclerotic plaque, specifically, at distances of 0.5 cm, 1 cm, and 1.5 cm from the carotid bifurcation. A cIMT greater than 0.9 mm in one or both carotid arteries was classified as increased. The thickest cIMT from both CCAs was termed the cIMT MAX. The PSV of the ICA was measured using pulsed-wave Doppler beyond the bifurcation in the ICA, near the jaw angle.

A plaque on the carotid artery was defined, according to the Mannheim Consensus, as a focal structure encroaching into the lumen by at least 0.5 mm or 50% of the surrounding IMT value, with a thickness exceeding 1.5 mm from the media–adventitia interface to the intima–lumen interface [17]. Carotid atherosclerosis was identified either by an increased cIMT of more than 0.9 mm or the presence of a plaque. Stenosis of the ICA was recognized based on the North American Symptomatic Carotid Endarterectomy Trial (NASCET) criteria when carotid ultrasonography showed an extracranial ICA stenosis of 50% or more [18,19].

For the PN, plaques in the common, bifurcation, and internal carotid artery segments were tallied. Scores were assigned as follows: 0 for no plaque, 1 for one plaque, 2 for two plaques, and 3 for three or more plaques. The PSS was determined based on bilateral carotid stenosis, similar to the quantitative stenosis grading for coronary artery luminal stenosis [20]. Scores were assigned as 0 for no stenosis or plaque, 1 for a stenosis rate of 0–49% (mild), 2 for 50–69% (moderate), and 3 for 70–99% (severe). The carotid artery plaque score (PS) was derived by summing the maximum thickness of each individual plaque in the bilateral carotid artery, irrespective of plaque length [21]. A high PS was defined as a PS of 5 or more [22].

### 2.5. Statistical Analysis

The baseline characteristics of the participants, divided into two groups by sex, were assessed through univariate and multivariate analyses. Continuous variables were expressed as mean values with standard deviation (SD), while categorical variables were represented as counts accompanied by percentages. A multiple linear regression analysis was utilized to determine the correlation between inflammatory markers and carotid atherosclerosis indices derived from carotid artery ultrasonography, adjusting for variables such as age, BMI, LDL, triglycerides, HbA1c, smoking habits, alcohol consumption, and physical activity. Plaque scores that did not adhere to the normality assumption were subjected to log transformation and were presented as median values with their interquartile ranges (IQRs).

To evaluate the independent relationship between carotid atherosclerosis and systemic inflammatory markers, multivariable logistic regression or multinomial logistic regression analyses were performed, accounting for variables like age, BMI, LDL, triglycerides, HbA1c, smoking, alcohol, and physical activity. Odds ratios (ORs) and their respective 95% confidence intervals (CIs) were calculated. The subgroups were divided based on the degree of stenosis using the PSS, and correlation analysis was performed between inflammatory and carotid atherosclerosis indices. The predictive ability of inflammatory markers for carotid atherosclerosis was examined using receiver operating characteristic (ROC) curve analysis and the area under the curve (AUC) metric. All statistical analyses were conducted using SPSS Software version 26.0 (IBM Co., Armonk, NY, USA) and MedCalc Version 20.023 (MedCalc Software Ltd., Ostend, Belgium). A *p*-value of less than 0.05 was deemed statistically significant.

## 3. Results

### 3.1. Baseline Characteristics

The study analyzed a total of 1264 participants, comprising 708 males and 556 females. The participants had a mean age of 56.15 years and an average BMI of 24.31 kg/m^2^. Although some subjects had comorbidities, they were well managed and within the normal range. Specifically, 294 (23.3%) had hypertension, 158 (12.5%) had diabetes, and 138 (10.9%) had dyslipidemia. In terms of lifestyle habits, 333 (26.3%) were non-drinkers and 668 (52.8%) were non-smokers. There was a notable difference in alcohol and smoking habits between genders. Among males, 626 (88.4%) consumed alcohol, 370 (52%) were former smokers, and 194 (27.4%) were current smokers. For females, 54.9% consumed alcohol, which is nearly the same proportion as non-drinkers at 45.1%. Additionally, 16 (2.9%) female participants were current smokers, 16 (2.9%) were former smokers, and a significant majority, 94.2%, had never smoked. When it comes to physical activity, 597 (47.2%) were categorized as non-active exercisers, while 667 (52.8%) were in the active group.

Laboratory results revealed that the average NLR for men was 1.82, and for women, it was 1.61. The PLR values were 135.89 for men and 150.38 for women. The hsCRP levels were 0.129 in males and 0.1 in females, and the ESR values stood at 9.91 for men and 14.79 for women. Notably, men had slightly elevated NLR and hsCRP levels, whereas women showed marginally higher PLR and ESR values (Table 1).

### 3.2. Carotid Atherosclerosis Indicators

The average cIMT for both the left and right CCAs was 0.77. For the ICA, the PSV was 64.22 on the right and 63.72 on the left.

Regarding the presence of plaque, 39.5% of men had plaque in the right carotid artery and 41.9% in the left, while 23.6% of women had plaque on the right side and 25.7% on the left. Luminal stenosis exceeding 50% of the ICA diameter was observed in five males on the right side, one male on the left, and one female on the right.

The study participants were further categorized based on the PN. There were 688 patients with no plaque (54.4%), 280 with one plaque (22.2%), 194 with two plaques (15.3%), and 102 with three or more plaques (8.1%). In terms of the PSS, 1142 patients showed no stenosis (90.3%), 115 had mild stenosis (9.1%), 6 had moderate stenosis (0.5%), and 1 exhibited severe stenosis (0.1%).

For the PS, the highest score recorded was approximately 20.7 for males and 10.7 for females (Table 2).

### 3.3. Inflammatory Markers and Carotid Atherosclerosis Indicators

The hsCRP demonstrated a notable correlation with the cIMT MAX and PN in the male group, as well as with the PS in both male and female groups (*p* < 0.05). The ESR showed a significant association with the cIMT MAX, PN, and PS in the male group. Both the NLR and PLR exhibited a significant relationship with the cIMT MAX, PN, and PS in both male and female groups (*p* < 0.05). Consequently, all four inflammatory markers (hsCRP, ESR, NLR, and PLR) were positively and significantly correlated with the cIMT, PN, and PS (*p* < 0.05) (Table 3). Following the exclusion of subjects without stenosis, carotid atherosclerosis indices and inflammatory markers were analyzed within subgroups comprising subjects with mild stenosis and those with moderate to severe stenosis. Significant correlations were identified between PSs and all inflammatory markers in both groups (Supplementary Tables S1 and S2).

### 3.4. NLR and PLR as Atherosclerosis Risk Factors

We further explored the NLR and PLR as potential risk factors for atherosclerosis using multivariable logistic regression and multinomial logistic regression (Table 4). For the NLR, the odds ratio (OR) for an increased cIMT (≥0.9 mm) was 1.15 (95% CI, 1.03–2.15; *p* = 0.032) for males and 1.05 (95% CI, 1.01–1.29; *p* = 0.009) for females. For the PLR, the OR was 1.41 (95% CI, 1.12–3.15; *p* = 0.041) for males and 1.53 (95% CI, 1.17–2.18; *p* = 0.002) for females.

As a risk factor for a high PS (≥5), the OR for the NLR was 1.35 (95% CI, 1.08–1.70; *p* = 0.008) for males and 1.68 (95% CI, 1.00–2.81; *p* = 0.049) for females. For the PLR, it was 1.65 (95% CI, 1.28–1.90; *p* < 0.001) for males and 1.91 (95% CI, 1.05–2.81; *p* = 0.032) for females.

Furthermore, the OR for the NLR as the risk for the presence of more than three plaques was 1.47 (95% CI, 1.03–2.61; *p* = 0.039) for males and 1.32 (95% CI, 1.07–3.25; *p* = 0.045) for females. For the PLR, it was 1.52 (95% CI, 1.09–2.41; *p* = 0.021) for males and 1.64 (95% CI, 1.10–2.63; *p* = 0.035) for females.

### 3.5. Evaluating Systemic Inflammatory Markers as Predictors of Carotid Atherosclerosis

To ascertain the potential of systemic inflammatory markers as predictors for carotid atherosclerosis, we employed the ROC curve analysis and documented the AUC. For cases with a PS of 5 or greater, the AUC for the NLR was recorded as 0.556 (95% CI: 0.517–0.596; *p* = 0.005) for males and 0.666 (95% CI: 0.542–0.790; *p* = 0.028) for females. Concurrently, the AUC for PLR was 0.597 (95% CI: 0.524–0.624; *p* = 0.002) for males and 0.635 (95% CI: 0.544–0.697; *p* = 0.019) for females. These results are further detailed in Table 5.

## 4. Discussion

This study sought to ascertain the relationship between systemic inflammatory markers and carotid atherosclerosis. Our findings indicate a significant correlation between the hsCRP, ESR, NLR, and PLR and the cIMT, PN, and PS among relatively healthy middle-aged individuals. Atherosclerosis is a chronic and progressive disease with stages of formation, progression, and propagation that take decades [23]. Inflammation is pivotal at all stages of atherosclerosis, being considered the primary pathophysiological factor in cardiovascular disease [24].

The ESR, CRP, NLR, and PLR are significant inflammatory markers and mediators. They are cost-effective, easily evaluated, and simple to test. The ESR and CRP are commonly included in routine serum blood tests. Meanwhile, the NLR and PLR can be readily calculated from a complete blood cell count. The role of the NLR and PLR as markers of inflammatory activity and their associations with adverse cardiovascular disease outcomes have been well-established in numerous previous studies [25,26,27,28]. A recent Korean investigation highlighted the potential of the NLR as an indicator of cardiovascular disease risk among prediabetic patients [29]. Prior research has unveiled links between the NLR and carotid artery stenosis [30], while another suggests that PLR could serve as both a conventional risk factor and a predictive biomarker for severe atherosclerosis [31]. Our findings are in line with previous research. In this study, the cIMT was positively correlated with inflammatory markers (CRP, ESR, NLR, and PLR) in men and with the NLR and PLR in women. The PN, except for the CRP in men, and the PS also showed positive associations with systemic inflammatory markers. The study observed a relationship between inflammatory markers and carotid indices, reaffirming the potential of the NLR and PLR as predictive tools. Both the NLR and PLR exhibited a positive correlation with increased cIMT, PS, and PN, solidifying their role in predicting carotid atherosclerosis.

Neutrophils discharge a plethora of active substances, including arachidonic acid metabolites, platelet aggravating factors, cytotoxic oxygen-derived free radicals, myeloperoxidase, elastase, and acid phosphatase. These play roles in mediating inflammatory responses, thereby contributing to plaque formation and vulnerability [32]. When platelets adhere to the endothelium, they signal monocytes to the inflammation site. The ensuing extravasation of monocytes and their transformation into macrophages mark the onset of atherosclerosis [33,34]. Conversely, lymphocytes, implicated in atherosclerosis pathogenesis, undergo apoptosis due to glucocorticoids secreted during stress from inflammation, intensifying atherosclerosis. Additionally, lymphocytes participate in anti-inflammatory processes and protect the endothelium [35]. Elevated NLR and PLR levels might indicate a systemic inflammatory state resulting from an imbalance among neutrophils, platelets, and lymphocytes. Consequently, the NLR and PLR may hold potential in reflecting the severity of plaque in atherosclerosis patients, offering insights into the broader scope of inflammatory activity.

In this study, the NLR and PLR showed a significant positive correlation with relatively increased cIMT, increased PS, and 3 or more plaque nodules in both men and women. And the NLR and PLR have the potential to be predictive indicators of carotid atherosclerosis. Although the value of ROC was low and the clinical value was not sufficient, it is considered to be a statistically significant indicator. This may be a result of the characteristics of the participants in this study. The study was conducted on relatively healthy participants, even if they had already been diagnosed with comorbidities that are traditional risk factors for cardiovascular disease, such as hypertension, dyslipidemia, and diabetes, but that were well controlled, and they had no history of ischemic heart disease or cerebrovascular events. In this study, 34.8% of participants had carotid plaques, while moderate (≥50%) and severe (≥70%) stenosis were observed in only 0.5% and 0.1% of cases, respectively. These percentages align reasonably with the prevalence range for moderate asymptomatic carotid stenosis (ACAS) (0% to 7.5%) and severe ACAS (0% to 3.1%) reported in general population studies [36]. The relatively modest degree of atherosclerosis in our sample suggests a weak correlation between ultrasound indices like the plaque stenosis score (PSS) and inflammatory markers, hence limiting the clinical applicability of these statistical findings. Nonetheless, our study is significant in establishing a relationship between ultrasound-measured carotid atherosclerosis indicators and systemic inflammatory markers. It implies that the limitations of ultrasound, chiefly its operator-dependent variability, can be complemented by blood tests as objective arteriosclerosis predictors, contributing to the prevention and management of cardiovascular disease (CVD). Still, more research is necessary to assess the clinical utility of these markers as screening tools.

This study has several limitations that warrant mention. First, its geographic focus on a single city in Korea limits the generalizability of the findings. While we established stringent inclusion and exclusion criteria to bolster the study’s power and assiduously selected subjects based on these criteria, the study’s scope remains localized. Efforts were made to include as many subjects as possible to mitigate this limitation. Second, the nature of ultrasonography as a real-time procedure introduces potential operator-dependent variability. To address this, examinations were conducted by well-trained radiologists using standardized methodologies. Lastly, the retrospective design of the study introduces the potential for biases. To minimize selection bias, individual authors independently reviewed medical records, rigorously applied selection criteria, and meticulously collected and reviewed data.

On the brighter side, this study offers notable strengths. We centered our research on a unique group of individuals: those without prior cardiovascular or cerebrovascular incidents and without uncontrolled health conditions, giving us insights into a segment of relatively healthy, active middle-aged individuals. Additionally, our approach to measuring carotid atherosclerosis was based on validated indices like the plaque score, plaque number score, and stenosis score, setting our study apart from many that focused merely on intima–media thickness (IMT) or the simple presence of plaque. Furthermore, the ease of deriving NLR and PLR values from a routine complete blood count (CBC) underscores their potential value. If proven to be reliable indicators of cardiovascular disease risk, their integration into clinical practices could be transformative, and our study serves as a stepping stone in this promising direction.

## 5. Conclusions

The NLR and PLR, derived from CBC and WBC differential counts, offer easy accessibility compared to other inflammatory markers. Their utilization also offers a cost-effective approach, benefiting patients financially. This study revealed notable correlations between these systemic inflammatory markers, especially the NLR and PLR, and indices of carotid atherosclerosis among relatively healthy middle-aged individuals. As such, the NLR and PLR may potentially serve as valuable surrogate markers of carotid atherosclerosis. Future large-scale prospective studies are warranted to validate their predictive value further.

## Figures and Tables

**Table 1:** Baseline characteristics.

	Total (*n* = 1264)	Male (*n* = 708)	Female (*n* = 556)	*p*-Value
Age, years	56.15 ± 6.31	55.92 ± 6.49	56.55 ± 5.98	0.136
Height, cm	166.18 ± 8.22	170.93 ± 5.61	158.27 ± 5.23	<0.001
Weight, cm	67.53 ± 11.47	73.04 ± 9.53	58.36 ± 8.06	<0.001
BMI, kg/m^2^	24.31 ± 3.11	24.91 ± 2.93	23.31 ± 3.15	0.039
SBP, mmHg	125.00 ± 15.84	125.76 ± 15.17	123.72 ± 16.85	0.045
DBP, mmHg	76.78 ± 11.35	78.16 ± 11.13	74.46 ± 11.36	0.011
Comorbidities				
Hypertension	294 (23.2)	170 (24.0)	124 (22.3)	0.009
Diabetes	158 (12.5)	100 (14.1)	58 (10.4)	<0.001
Dyslipidemia	138 (10.9)	65 (9.2)	73 (13.1)	<0.001
Alcohol *				<0.001
None	333 (26.3)	82 (11.6)	251 (45.1)	
Drinker	931 (73.7)	626 (88.4)	305 (54.9)	
Smoking				<0.001
None	668 (52.8)	144 (20.3)	524 (94.2)	
Former	386 (30.5)	370 (52.3)	16 (2.9)	
Current	210 (16.6)	194 (27.4)	16 (2.9)	
Physical activity **				<0.001
None	597 (47.2)	320 (45.2)	277 (49.8)	
Regular	667 (52.8)	388 (54.8)	279 (50.2)	
Total cholesterol	191.86 ± 40.12	187.39 ± 39.82	199.32 ± 39.54	<0.001
Triglyceride	127.84 ± 88.68	143.63 ± 98.18	101.46 ± 61.61	<0.001
LDL-cholesterol	130.45 ± 38.52	127.52 ± 37.62	135.36 ± 39.52	<0.001
HDL-cholesterol	56.63 ± 14.48	52.42 ± 12.38	63.67 ± 15.00	<0.001
FBS	108.13 ± 24.67	111.87 ± 25.48	103.54 ± 22.54	<0.001
HbA1c	5.72 ± 0.76	5.75 ± 0.80	5.67 ± 0.68	<0.001
NLR	1.74 ± 0.75	1.82 ± 0.77	1.61 ± 0.68	<0.001
PLR	141.53 48.10	135.89 ± 45.50	150.38 ± 50.71	<0.001
hsCRP	0.118 ± 0.270	0.129 ± 0.316	0.100 ± 0.168	0.015
ESR	11.75 ± 9.73	9.91 ± 8.72	14.79 ± 10.52	<0.001

All values are presented as the mean ± standard deviation or *n* (%); the baseline characteristics of the two groups were analyzed by independent *t*-test or chi-square test. * Drinker was defined as a participant who takes 14 standard drinks (male) or 7 standard drinks (female) per week. ** Active was defined as a participant who exercises more than 150 min per week. BMI, body mass index; SBP, systolic blood pressure; DBP, diastolic blood pressure; LDL, low-density lipoprotein; HDL, high-density lipoprotein; FBS, fasting blood sugar; HbA1c, hemoglobin A1c; NLR, neutrophil–lymphocyte ratio; PLR platelet-to-lymphocyte ratio; hsCRP, high-sensitivity C-reactive protein; ESR, erythrocyte sedimentation rate.

**Table 2:** Carotid atherosclerosis indices of participants.

	Total (*n* = 1264)	Male (*n* = 708)	Female (*n* = 556)
cIMT			
Right, mm	0.77 ± 0.16	0.77 ± 0.17	0.75 ± 0.16
Left, mm	0.77 ± 0.17	0.78 ± 0.17	0.75 ± 0.15
PSV			
Right ICA, cm/s	64.22 ± 16.89	60.96 ± 15.49	69.67 ± 17.71
Left ICA, cm/s	63.72 ± 17.56	60.55 ± 16.73	69.02 ± 17.66
Plaque on CA			
Right	411 (32.5)	280 (39.5)	131 (23.6)
Left	440 (34.8)	297 (41.9)	143 (25.7)
Stenosis *			
Right ICA	6 (0.5)	5 (0.7)	1 (0.2)
Left ICA	1 (0.1)	1 (0.1)	0 (0.0)
Plaque number score (PN)			
0	688 (54.4)	331 (46.8)	357 (64.2)
1	280 (22.2)	166 (23.4)	114 (20.5)
2	194 (15.3)	129 (18.2)	65 (11.7)
>3	102 (8.1)	82 (11.6)	20 (3.6)
Plaque stenosis score (PSS)			
0	1142 (90.3)	610 (86.2)	532 (95.7)
1	115 (9.1)	94 (13.2)	21 (3.7)
2	6 (0.5)	4 (0.6)	2 (0.4)
3	1 (0.1)	0 (0.0)	1 (0.2)
Carotid artery plaque score (PS) **	1.53 ± 2.30 (min 0.00, max 20.70)	1.91 ± 2.59 (min 0.00, max 20.70)	0.89 ± 1.52 (min 0.00, max 10.70)

All values were presented as the mean ± standard deviation or *n* (%); * Carotid artery luminal stenosis ≥50% by NASCET; ** Carotid artery plaque scores (PSs) were log-transformed and were presented as median ± interquartile range; cIMT, carotid intima–media thickness; PSV, peak systolic velocity; ICA, internal carotid artery; CA, carotid artery.

**Table 3:** Association of carotid atherosclerosis indices with systemic inflammatory markers.

		hsCRP			ESR			NLR			PLR	
	β (S.E.)	R^2^	*p*-Value	β (S.E.)	R^2^	*p*-Value	β (S.E.)	R^2^	*p*-Value	β (S.E.)	R^2^	*p*-Value
cIMT MAX *												
Male	0.014 (0.019)	0.435	0.025	0.002 (0.001)	0.482	0.001	0.013 (0.010)	0.555	<0.001	0.025 (0.017)	0.562	0.001
Female	−0.011 (0.040)	0.521	0.793	0.001 (0.001)	0.452	0.085	0.010 (0.010)	0.525	0.038	0.035 (0.013)	0.501	0.009
Plaque number score (PN)												
Men	0.179 (0.113)	0.652	0.048	0.020 (0.004)	0.399	<0.001	0.023 (0.045)	0.321	0.041	0.134 (0.030)	0.589	0.021
Female	0.313 (0.224)	0.555	0.163	0.010 (0.003)	0.378	0.004	0.020 (0.053)	0.371	0.046	0.149 (0.045)	0.600	0.018
Plaque stenosis score (PSS)												
Men	−0.022 (0.014)	0.351	0.585	0.001 (0.029)	0.513	0.383	0.013 (0.016)	0.557	0.422	0.002 (0.001)	0.458	0.651
Female	0.049 (0.068)	0.306	0.477	0.001 (0.001)	0.492	0.365	0.023 (0.016)	0.528	0.356	0.009 (0.001)	0.364	0.664
Plaque score (PS)												
Men	0.085 (0.035)	0.685	0.012	0.010 (0.006)	0.625	0.001	0.034 (0.022)	0.699	<0.001	0.055 (0.033)	0.612	0.015
Female	0.058 (0.026)	0.565	0.037	0.009 (0.003)	0.619	0.002	0.025 (0.013)	0.682	0.015	0.070 (0.059)	0.558	0.023

We used multiple linear regression analysis using inflammatory markers and scores from ultrasonography. Since plaque scores did not show normal distribution, they were standardized through log transformation. Adjusted for age, body mass index, LDL, TG, HbA1c, smoking, alcohol, and physical activity; * cIMT MAX refers to the thickness at the thickest intima of the common carotid artery on both sides. cIMT, common carotid artery intima–media thickness; PN, plaque number score; PSS, plaque stenosis score; PS, plaque score.

**Table 4:** Odds ratio of neutrophil–lymphocyte ratio and platelet–lymphocyte ratio as risk factors for carotid atherosclerosis.

**NLR**				
	**Male**		**Female**	
	**Odds Ratio (95% CI)**	***p*** -Value *	**Odds Ratio (95% CI)**	***p*** **-Value**
cIMT (≥0.9)	1.15 (1.03–2.15)	0.032	1.05 (1.01–1.29)	0.009
PS (≥5)	1.35 (1.08–1.70)	0.008	1.68 (1.00–2.81)	0.049
PN				
0	1 (Ref)		1 (Ref)	
1	1.01 (0.69–1.96)	0.152	1.02 (0.51–1.64)	0.352
2	1.09 (1.01–1.85)	0.047	1.05 (0.85–2.66)	0.139
3	1.47 (1.03–2.61)	0.039	1.32 (1.07–3.25)	0.045
**PLR**				
	**Male**		**Female**	
	**Odds Ratio (95% CI)**	***p*** -Value *	**Odds Ratio (95% CI)**	***p*** **-Value**
cIMT (≥0.9)	1.41 (1.12–3.15)	0.041	1.53 (1.17–2.18)	0.002
PS (≥5)	1.65 (1.28–1.90)	<0.001	1.91 (1.05–2.81)	0.032
PN				
0	1 (Ref)		1 (Ref)	
1	1.11 (0.51–1.36)	0.121	1.19 (0.51–1.92)	0.446
2	1.25 (0.99–1.92)	0.054	1.24 (0.95–2.51)	0.099
3	1.52 (1.09–2.41)	0.021	1.64 (1.10–2.63)	0.035

* *p*-values were calculated by multivariable logistic regression analysis or multinomial logistic regression analysis covarying for age, BMI, LDL, TG, HbA1c, smoking, alcohol, and physical activity. CI, confidence interval; cIMT, carotid intima–media thickness; PS, plaque score; PN, plaque number score.

**Table 5:** Receiver operating characteristic curve analysis of neutrophil–lymphocyte ratio and platelet–lymphocyte ratio as predictors for carotid atherosclerosis.

	Male		Female	
	AUC (95% CI)	*p*-Value	AUC (95% CI)	*p*-Value
cIMT (≥0.9)				
hsCRP	0.518 (0.478–0.559)	0.382	0.519 (0.463–0.575)	0.509
ESR	0.541 (0.482–0.599)	0.178	0.545 (0.490–0.599)	0.115
NLR	0.518 (0.479–0.556)	0.381	0.450 (0.395–0.504)	0.072
PLR	0.557 (0.439–0.612)	0.189	0.525 (0.475–0.575)	0.125
PN (≥1)				
hsCRP	0.529 (0.491–0.567)	0.140	0.561 (0.510–0.613)	0.019
ESR	0.569 (0.532–0.606)	<0.001	0.542 (0.492–0.592)	0.107
NLR	0.488 (0.451–0.526)	0.537	0.508 (0.458–0.558)	0.752
PLR	0.531 (0.476–0.571)	0.338	0.527 (0.485–0.563)	0.215
PS (≥5)				
hsCRP	0.541 (0.482–0.599)	0.178	0.468 (0.328–0.609)	0.674
ESR	0.601 (0.540–0.662)	0.001	0.567 (0.446–0.689)	0.375
NLR	0.556 (0.517–0.596)	0.005	0.666 (0.542–0.790)	0.028
PLR	0.597 (0.524–0.624)	0.002	0.635 (0.544–0.697)	0.019

AUC, area under curve; CI, confidence interval; cIMT, carotid intima–media thickness; hsCRP, high-sensitivity C-reactive protein; ESR, erythrocyte sedimentation rate; NLR, neutrophil–lymphocyte ratio; PN, plaque number score; PS, plaque score.

## Data Availability

Data that are not presented in the article are available upon reasonable request from the corresponding author.

## Supplementary Materials

The following supporting information can be downloaded at: https://www.mdpi.com/article/10.3390/jcdd11030073/s1, Table S1: Association of Carotid atherosclerosis indices with systemic inflammatory markers in subjects with mild stenosis; Table S2: Association of Carotid atherosclerosis indices with systemic inflammatory markers in subjects with moderate to severe stenosis
