# Supplementary material for: Exploring the Correlation between Systemic Inflammatory Markers and Carotid Atherosclerosis Indices in Middle-Aged Adults: A Cross-Sectional Study
Source: J Cardiovasc Dev Dis. 2024 Feb 21;11(3):73. doi: 10.3390/jcdd11030073 (PMC10971297; doi:10.3390/jcdd11030073)
Supplement: Supplementary file 1 [file jcdd-11-00073-s001.zip › jcdd-2719798-supplementary.pdf]

Supplementary Table S1. Association of Carotid atherosclerosis indices with systemic inflammatory markers in subjects with mild stenosis

	Hs-CRP		ESR		NLR		PLR	
	r*	P-value	r	P-value	r	P-value	r	P-value
cIMT Max**	0.32	0.035	0.20	0.069	0.44	0.039	0.57	0.042
Plaque Number Score (PN)	0.10	0.857	0.09	0.225	0.40	0.041	0.45	0.008
Plaque score (PS)	0.52	0.020	0.28	0.010	0.58	0.029	0.55	0.005

We used correlation analysis using inflammatory markers and scores from ultrasonography. Since plaque scores did not show normal distribution, they are standardized through log transformation. Adjusted for age, body mass index, LDL, TG, HbA1c, smoking, alcohol, and physical activity

* Pearson correlation coefficient

**cIMT Max refers to the thickness at the thickest intima of the common carotid artery on both sides. cIMT, common carotid artery intima-media thickness; PSV, peak systolic velocity; ICA, internal carotid artery; CCA, common carotid artery; PN, plaque number score; PSS, plaque stenosis score; PS, plaque score

Supplementary Table S2. Association of Carotid atherosclerosis indices with systemic inflammatory markers in subjects with moderate to severe stenosis

	Hs-CRP		ESR		NLR		PLR	
	r*	P-value	r	P-value	r	P-value	R	P-value
cIMT Max**	0.35	0.048	0.23	0.044	0.55	0.028	0.61	0.011
Plaque Number Score (PN)	0.05	0.665	0.15	0.107	0.48	0.049	0.53	0.035
Plaque score (PS)	0.62	0.041	0.31	0.025	0.60	0.016	0.69	0.009

We used correlation analysis using inflammatory markers and scores from ultrasonography.
Adjusted for age, body mass index, LDL, TG, HbA1c, smoking, alcohol, and physical activity

* Spearman correlation coefficient

**cIMT Max refers to the thickness at the thickest intima of the common carotid artery on both sides. cIMT, common carotid artery intima-media thickness; PSV, peak systolic velocity; ICA, internal carotid artery; CCA, common carotid artery; PN, plaque number score; PSS, plaque stenosis score; PS, plaque score
